# Supplementary material for: Identification of key sectors of water resource utilization in China from the perspective of water footprint
Source: PLoS One. 2020 Jun 22;15(6):e0234307. doi: 10.1371/journal.pone.0234307 (PMC7307777; doi:10.1371/journal.pone.0234307)
Supplement: S1 Appendix — (DOC) [file pone.0234307.s001.doc]

Appendix: Sector List

The sectors are as follows ( compared to 123 sectors in the Eora database, Sector 5, Sector 95-97 and Sector 99 in this paper are formed by merge.)

| **Code** | **Sector name** |
| --- | --- |
| S1 | Crop cultivation |
| S2 | Forestry |
| S3 | Logging and transport of timber and bamboo |
| S4 | Livestock and livestock products |
| S5 | Fishery,technical services for agriculture, forestry, livestock and fishing |
| S6 | Coal mining and processing |
| S7 | Crude petroleum products and Natural gas products |
| S8 | Ferrous ore mining |
| S9 | Non-ferrous ore mining |
| S10 | Salt mining |
| S11 | Non-metal minerals and other mining |
| S12 | Grain mill products |
| S13 | Feeding stuff production and processing |
| S14 | Vegetable oil and forage |
| S15 | Sugar refining |
| S16 | Slaughtering, meat processing,eggs and dairy products |
| S17 | Prepared fish and seafood |
| S18 | Other food products |
| S19 | Wines, spirits and liquors |
| S20 | Non-alcoholic beverage |
| S21 | Tobacco products |
| S22 | Cotton textiles |
| S23 | Woolen textiles |
| S24 | Hemp textiles |
| S25 | Other textiles not elsewhere classified |
| S26 | Knitted mill |
| S27 | Wearing appare |
| S28 | Leather, furs, down and related products |
| S29 | Sawmills and fibreboard |
| S30 | Furniture and products of wood, bamboo, cane, palm, straw, etc. |
| S31 | Paper and products |
| S32 | Printing and record medium reproduction |
| S33 | Cultural goods |
| S34 | Toys, sporting and athletic and recreation products |
| S35 | Petroleum refining |
| S36 | Coking |
| S37 | Raw chemical materials |
| S38 | Chemical fertilizers |
| S39 | Chemical pesticides |
| S40 | Chemicals for painting, dying and others |
| S41 | Synthetic chemicals |
| S42 | Chemicals for special usages |
| S43 | Chemical products for daily use |
| S44 | Medical and pharmaceutical products |
| S45 | Chemical fibers |
| S46 | Rubber products |
| S47 | Plastic products |
| S48 | Cement and cement asbestos products |
| S49 | Glass and glass products |
| S50 | Pottery, china and earthenware |
| S51 | Fireproof products |
| S52 | Other non-metallic mineral products |
| S53 | Iron-smelting |
| S54 | Steel-smelting |
| S55 | Steel-processing |
| S56 | Alloy iron smelting |
| S57 | Nonferrous metal smelting |
| S58 | Nonferrous metal processing |
| S59 | Metal products |
| S60 | Boiler, engines and turbine |
| S61 | Metalworking machinery |
| S62 | Other general industrial machinery |
| S63 | Agriculture, forestry, animal husbandry and fishing machinery |
| S64 | Other special industrial equipment |
| S65 | Railroad transport equipment |
| S66 | Motor vehicles |
| S67 | Vehicles fittings production |
| S68 | Ship building |
| S69 | Other transport machinery |
| S70 | Generators |
| S71 | Household electric appliances |
| S72 | Other electric machinery and equipment |
| S73 | Communication equipment |
| S74 | Electronic computer |
| S75 | Other computer devices |
| S76 | Electronic element and device |
| S77 | Electronic appliances |
| S78 | Other electronic and communication equipment |
| S79 | Instruments, meters and other measuring equipment |
| S80 | Cultural and office equipment |
| S81 | Arts and crafts products |
| S82 | Other manufacturing products |
| S83 | Scrap and waste |
| S84 | Electricity and steam production and supply |
| S85 | Gas production and supply |
| S86 | Water production and supply |
| S87 | Construction |
| S88 | Railway passenger transport |
| S89 | Railway freight transport |
| S90 | Highway freight and passengers transport |
| S91 | Domestic public transport |
| S92 | Water freight and passengers transport |
| S93 | Air passenger transport |
| S94 | Air freight transpor |
| S95 | Pipeline transport, Warehousing, Post, Telecommunication, Computing services and software, Wholesale and retail trade |
| S96 | Hotels, Eating and drinking places, Finance, Insurance, Real estate, Leasehold, Business services, Tourism, Scientific research, General technical services, Geological prospecting |
| S97 | Water conservancy, Environmental resources and public infrastructure, Resident services and other services |
| S98 | Educational services |
| S99 | Health services, Social welfare, Culture and arts, radio, film and television, Sports, Recreational services, Public administration and other sectors, Re-export & Re-import |
