# Supplementary material for: Identification of key sectors of water resource utilization in China from the perspective of water footprint
Source: PLoS One. 2020 Jun 22;15(6):e0234307. doi: 10.1371/journal.pone.0234307 (PMC7307777; doi:10.1371/journal.pone.0234307)
Supplement: Attachment — Submitted filename: Comments.docx [file pone.0234307.s002.docx]

**Comments**

**The paper studied the China’s water by drawing on the model from Alcantara and Padilla[7]. Furthermore, the authors spent much time collecting data and writing the paper. However, the authors did not show that why the subject was selected and what problem and question of China’s water the paper would be addressed. There are some problems which would be corrected by the authors as follows:**

**1.Writing skill in English should be improved,Chinese characters can even be found in this paper.**

**2.Pay attention to the format, for example,”Chinafrom”,”footprintand”.(Page3,Line 14)**

**3.I suggest Figure 2 be more clear and beautiful to facilitate the readers.**

**4.The logic of literature review is a little confused. Are there no relevant researches on identification of key sectors of water resource utilization in China? What is the innovation of this paper?**

**5.The author employs 0.003 as the threshold to classify all sectors in this paper. However, the valid reasons why is 0.003 suitable fail to be provided.**

**6.The author mainly describes the measurement results in this paper. But further discussions on this results find no appearance.**

**7.The author mentions that Chinese government should design water-saving policies and control the economic growth of key sectors.However,concrete methods also can not be found.**
