# Supplementary material for: Identification of key sectors of water resource utilization in China from the perspective of water footprint
Source: PLoS One. 2020 Jun 22;15(6):e0234307. doi: 10.1371/journal.pone.0234307 (PMC7307777; doi:10.1371/journal.pone.0234307)
Supplement: Attachment — Submitted filename: Response.docx [file pone.0234307.s003.docx]

Dear Chuanwang Sun

Thank you very much for giving us an opportunity to revise our manuscript. We appreciate the editor and reviewers very much for their constructive comments and suggestions on our manuscript entitled “Identification of key sectors of water resource utilization in China from the perspective of the water footprint” (PONE-D-19-27097)

**We have studied reviewers’ comments carefully. According to the reviewers’ detailed suggestions, we have made a careful revision on the original manuscript. All revised portions are marked in red in the revised manuscript which we would like to submit for your kind consideration.**

Funding Statement: This work was supported by the Natural Science Foundation of China under Grant [number 71704070]; Ministry of Education for the Humanities and Social Sciences Research Young Fund on the West and Borderland Project [number 17XJC790002]; Guangdong Provincial Natural Science Foundation of China under Grant [number 2017A030313443]; and Program of Lanzhou University of Finance and Economics under Grant [number Lzufe2018B-06].

**Kind regards**

Guangyao Deng

Email:[dgy316203@163.com](mailto:dgy316203@163.com)

Corresponding author : Xiaofang Yue

E-mail address: harborangel@163.com

Dear reviewers:

**Thank you for your comments on our manuscript entitled** “Identification of key sectors of water resource utilization in China from the perspective of the water footprint” (PONE-D-19-27097). **Those comments are very helpful for revising and improving our paper, as well as the important guiding significance to other research. We have studied the comments carefully and made corrections which we hope meet with approval. The main corrections are in the manuscript and the responds to the reviewers’ comments are as follows (the replies are highlighted in red ).**

Journal Requirements:

When submitting your revision, we need you to address these additional requirements.

1. Please ensure that your manuscript meets PLOS ONE's style requirements, including those for file naming. The PLOS ONE style templates can be found at http://www.journals.plos.org/plosone/s/file?id=wjVg/PLOSOne_formatting_sample_main_body.pdf and http://www.journals.plos.org/plosone/s/file?id=ba62/PLOSOne_formatting_sample_title_authors_affiliations.pdf

Response:This revision is made by referring to the format of PLOS ONE.

2. PLOS requires an ORCID iD for the corresponding author in Editorial Manager on papers submitted after December 6th, 2016. Please ensure that you have an ORCID iD and that it is validated in Editorial Manager. To do this, go to ‘Update my Information’ (in the upper left-hand corner of the main menu), and click on the Fetch/Validate link next to the ORCID field. This will take you to the ORCID site and allow you to create a new iD or authenticate a pre-existing iD in Editorial Manager. Please see the following video for instructions on linking an ORCID iD to your Editorial Manager account: <https://www.youtube.com/watch?v=_xcclfuvtxQ>

Response:The corresponding author has registered an ORCID iD.

Xiaofang Yue

https://orcid.org/0000-0002-4773-7670

3.  Thank you for stating the following in the Acknowledgments Section of your manuscript:

'This work was supported by the Natural Science Foundation of China under
Grant [number 71704070]; Ministry of Education for the Humanities and Social Sciences
Research Young Fund on the West and Borderland Project [number 17XJC790002], Gansu
Provincial Higher Education Research Project [number 2017B-41]; Guangdong Provincial Natural
Science Foundation of China under Grant [number 2017A030313443];Program of Lanzhou
University ofFinance and Economics under Grant [number Lzufe2018B-06]; and the Research
Project of Institute of Silk RoadEconomics, Lanzhou University of Finance and Economics under
Grant[number JYYZ201603].'

We note that you have provided funding information that is not currently declared in your Funding Statement. However, funding information should not appear in the Acknowledgments section or other areas of your manuscript. We will only publish funding information present in the Funding Statement section of the online submission form.

Please remove any funding-related text from the manuscript and let us know how you would like to update your Funding Statement. Currently, your Funding Statement reads as follows:

'Yes'

Response: the funding-related text has been removed from the revision and moved to the Funding Statement.

4. Please upload a copy of Figure 3, to which you refer in your text on page 21. If the figure is no longer to be included as part of the submission please remove all reference to it within the text.

Response:We made an error in the original manuscript, Figure 3 is actually Figure 2, we have corrected it in the revision.

Additional Editor Comments (if provided):

[Note: HTML markup is below. Please do not edit.]

Reviewers' comments:

Reviewer #1: The paper studied the China’s water by drawing on the model from Alcantara and Padilla[7]. Furthermore, the authors spent much time collecting data and writing the paper.

However, the authors did not show that why the subject was selected and what problem and question of China’s water the paper would be addressed.

Response: The purpose of this paper is as follows: since the contradiction between the supply and demand of water resources in China is relatively prominent, it is very important to allocate the limited water resources reasonably. There are multiple sectors in China's economic system, and identifying key sectors of water use can help rationalize water resources. The key sector of water use defined in this paper is not only the sector that USES more water, but also the sector where water resource input can greatly promote economic growth. Therefore, this paper draws on the methods of Alcantara and Padilla[7] for reference and combines the water resource use data in EORA database to identify the key sectors of water resource use in China.

There are some problems which would be corrected by the authors as follows:
1.Writing skill in English should be improved,Chinese characters can even be found in this paper.

Response: We read the manuscript carefully, and with the help of native-speaker colleagues, we have revised the English language.

2.Pay attention to the format, for example,”Chinafrom”,”footprintand”.(Page3,Line 14)

Response: We are sorry that we made such a low-level mistake. We did not take into account that such problems would occur in the translation of different versions of Word documents. Such problems have been revised in the revision.

3.I suggest Figure 2 be more clear and beautiful to facilitate the readers.

Response: the revised figure is as follows (enlarged box and number)

4.The logic of literature review is a little confused. Are there no relevant researches on identification of key sectors of water resource utilization in China? What is the innovation of this paper?

Response: Existing literature on water footprint focuses on its accounting, which can only identify the key sectors that use more water, but cannot identify the sectors where water resources input can greatly drive economic growth. The innovation of this paper is to identify the key sectors of water resources use in terms of the amount of water used and the economic function of that water use by drawing on the method proposed by Alcantara and Padilla(2006) ,which is used to identify the key sectors of carbon emissions. It should be pointed out that the purpose of this paper is to identify the key sectors of China's water use in combination with water use and economic functions, not to account for the green, blue and grey water footprints of various sectors in China.

5.The author employs 0.003 as the threshold to classify all sectors in this paper. However, the valid reasons why is 0.003 suitable fail to be provided.

Response：Alcántara and Padilla (2003), Alcántara and Padilla (2006), Othman and Jafari (2016) and other literatures have employed median as the classification standard..Considering the characteristic that China's water use is mainly concentrated in the sector Crop cultivation (sector1), we employ 0.003 as the threshold for classification, which has already ensured that the total impact (TI) and direct impact (DI) of key sectors account for more than 50% of that of various sectors. In addition, since the input-output table used in this paper has 99 sectors, the number of key sectors identified by the median as a threshold will be too much.

6. The author mainly describes the measurement results in this paper. But further discussions on this results find no appearance.

Response: The following content is the discussion part of this paper.

In recent years, with the rapid development of economy, the contradiction between supply and demand of resources in China is becoming more and more serious, and environmental pressure is also growing. At present, a lot of research has been done to explore the problem of environmental pollution in China from the perspective of carbon footprint [35]-[40].This paper explores the contradiction between supply and demand of water resources in China from the perspective of water footprint.

Since the raw data collected from different researchers is not quite the same, there are different accounting results of China’s water footprint even with the same calculation method. For example, there are differences in terms of water footprint accounting results between this paper and existing literature on studies of water footprint for each sector in China[2]-[3][17]-[19][21], and there are differences among the literature itself. In the literature water footprint accounting in each region of China is based on the multi-region input-output model [1] [25]-[27][32], the sum of water footprint in each region is not equal to China’s water footprint in this paper. Additionally, the focus of most literature on studies of China’s water footprint with input-output model is green water footprint and blue water footprint, while only a few [17] are about these three altogether: China’s green water, blue water and grey water footprint.

Several studies [2][21] stressed the impact of the virtual water trade and water footprint, but the focus of this paper is the analysis of the key sectors of the water resource consumption in China. Based on the cross-elasticity of the value added with the water consumption, we defined the DI indicator and TI indicator as methods to identify key sectors of the water consumption instead of considering the amount of water used for each sector alone. Therefore, not only do the key sectors identified in this paper require a large amount of water, but their economic growth also has a relatively significant impact on the water resource use of other sectors. Table 4 shows that the direct impact dominant sectors of China’s water consumption in 2015 are Vegetable Oil and Forage (S14), Slaughtering, Meat Processing, Eggs, and Dairy Products(S16), Prepared Fish and Seafood(S17), and Arts and Crafts Products(S81). Though these sectors used a relatively large amount of water (DI>0.003), the impact of their economic growth on the water consumption of other sectors was comparatively small (TI<0.003). Therefore, they were not ranked as key sectors of China’s water consumption in 2015.

In terms of the sum of green water, blue water and grey water footprint, key sectors of China’s water consumption in 2015 are Crop cultivation(S1), Forestry(S2), Livestock and livestock products(S4), Fishery, Technical services for agriculture, forestry, livestock and fishing(S5), Other food products(S8), Scrap and waste(S83). These sectors belong to agriculture, food processing industry and wastewater treatment industry. Farming consumes a huge amount of water and farm products are necessary raw materials for production in other industries. Therefore, the Direct Impact (DI) indicator and Total Impact (TI) indicator are relatively great for Crop cultivation (S1), Forestry (S2), Livestock and livestock products (S4), Fishery, Technical services for agriculture, forestry, livestock and fishing (S5). As food processing industry is closely related to farm products, Direct Impact (DI) and Total Impact (TI) for Other food products (S18) are relatively great as well. Wastewater treatment industry mainly involves grey water footprint. To purify wastewater, lots of water is required. On the other hand, as production of most sectors brings about wastewater, wastewater treatment can’t be escaped. So the pull impact of increase in the value added for Scrap and waste (S83) on water consumption of other industries is relatively great. These explanations apply to the results concluded through Table 3 and Table 9, so they won’t be covered again.

Key sectors of China’s water use might vary from year to year. For instance, according to table 2, for sector S2 (Forestry), DI indicator in 2011-2013 is less than 0.003, which does not conform to TI>0.003 and DI>0.003, so it can’t be identified as a key sector. However, in 2014-2015, sector S2 (Forestry) meets TI>0.003 and DI>0.003, and thus it is identified as a key sector. In EORA database, water usage of each sector in China varies from year to year, and there are different input-output tables each year. Therefore, even for the same sector, DI indicator and TI indicator vary from year to year. Accordingly, key sectors of China’s water use might change in different years.

Key sectors of China’s water consumption might also change when it comes to different types of water footprint. For instance, according to Table 5 and Table 6, key sectors which use blue water are different from those with grey water. As the functions of blue water and grey water are not the same, one for production and services of each sector while the other for removing pollutant from water, sectors that use more blue water do not necessarily consume more grey water. In addition, how blue water use of other sectors changes, caused by the change in the value added of each sector, is different from the change in grey water use.

7.The author mentions that Chinese government should design water-saving policies and control the economic growth of key sectors.However,concrete methods also can not be found.

Response：The revision has added the following parts: In order to alleviate the growing contradiction between water supply and demand in China, the paper offers some strategies and measures that may help address the challenges, which include: (1) For industrial and agricultural water use, a system of applying for water, quota water supply, paid water supply, over-quota controlling or double charging can be implemented. (2) In areas where water resources are scarce, low-water-consuming industries should be developed and water- recycling systems should be adopted. (3) Strategies such as developing water-saving agriculture, preventing flood irrigation, reducing canal leakage, popularizing water-saving technologies (such as sprinkler irrigation, drip irrigation, and infiltration irrigation) should be taken.

Reviewer #2: It is interesting to analyze water resource utilization from the perspective of water footprint. It is better for the authors to conduct MAJOR REVISION.

The comments are as follows.
1. what is water footprint? why did the authors analyze from the perspective of water footprint? It is better to clarify in detail.

Response：The water footprint refers to the sum of the direct and indirect water use by a country (region or individual) for production (consumption)[4][10]-[11]。Since the production of each sector’s products requires not only direct input of water resources, but also products of other sectors as intermediate inputs, production of which also require water resources. This part of water can be regarded as indirect water use. So water resources use includes direct and indirect use. Therefore, the key sectors of water resources use need to be identified from the perspective of water footprint.

2. What is the motivation of this paper?

Response: Since the contradiction between the supply and demand of water resources in China is relatively prominent, it is very important to allocate the limited water resources reasonably. There are multiple sectors in China's economic system, and identifying key sectors of water use can help rationalize water resources. The key sector of water use defined in this paper is not only the sector that USES more water, but also the sector where water resource input can greatly promote economic growth. Therefore, this paper draws on the methods of Alcantara and Padilla[7] for reference and combines the water resource use data in EORA database to identify the key sectors of water resource use in China.

3. Compared with previous studies, what are the novelties of this paper?

Response：Existing literature on water footprint focuses on it’s accounting , which can only identify the key sectors that use more water, but cannot identify the sectors where water resources input can greatly drive economic growth. The innovation of this paper is to identify the key sectors of water resources use in terms of the amount of water used and the economic function of that water use by drawing on the method proposed by Alcantara and Padilla(2006) ,which is used to identify the key sectors of carbon emissions. It should be pointed out that the purpose of this paper is to identify the key sectors of China's water use in combination with water use and economic functions, not to account for the green, blue and grey water footprints of various sectors in China.

4. In literature reviews, it is better for the author to compare the previous papers on water resource and other resource such coal, gas and others such as:
[1] Decoupling CO2 emissions from economic growth in agricultural sector across 30 Chinese provinces from 1997 to 2014. Journal of Cleaner Production, Volume 159, 15 August 2017, Pages 220-228
[2] Nonrenewable energy, renewable energy, carbon dioxide emissions and economic growth in China from 1952 to 2012. Renewable and sustainable energy reviews, ,2015, 52：680-688.
[3] The Comparison Analysis of Total Factor Productivity and Eco-efficiency in China’s Cement Manufactures. Energy Policy. 81:61-66,,2015
[4] Are Stronger Environmental Regulations Effective in Practice? The Case of China’s Accession to the WTO , Journal of Cleaner Production , (39):161-167，2013.
[10] The influencing factors of CO2 emission intensity of Chinese agriculture from 1997 to 2014, Environmental Science and Pollution Research,2018， 25:13093–13101
[5] Different impacts of export and import on carbon emissions across 7 ASEAN countries: A panel quantile regression approach，Science of the Total Environment，2019，686, 1019-1029

Response: Thank you for your constructive suggestions. We have introduced these papers in the further discussion section.

In recent years, with the rapid development of economy, the contradiction between supply and demand of resources in China is becoming more and more serious, and environmental pressure is also growing. At present, a lot of research has been done to explore the problem of environmental pollution in China from the perspective of carbon footprint [35]-[40]. This paper explores the contradiction between supply and demand of water resources in China from the perspective of water footprint.

5.what are the differences among green water, blue water and grey water. It is better to introduce in detail.

Response: Green water refers to the water stored in the soil at the root of crops (mainly rain water), blue water refers to rivers, lakes, and groundwater with economic value, and grey water refers to the water needed to purify pollutants in the water(Hoekstra et al.,2011).

Hoekstra AY, Chapagain A K, Aldaya M M, Mekonnen M M(2011)The water footprint assessment manual: Setting the global standard. Routledge.

6.The paper also compare different industry sectors. what are the differences?

Response: There is a large difference in the amount of green water, blue water and gray water used in different industries, and the impact of water resources input on economic growth in various industries also varies greatly.

The empirical results from our a comprehensive analysis of the use of green water, blue water, and grey water show that the key sectors for water consumption in China in 2015 were Crop Cultivation (S1), Forestry (S2), Livestock and Livestock Products (S4), Fishery, Technical Services for Agriculture, Forestry, Livestock, and Fishing (S5), Other Food Products (S18), and Scrap and Waste (S83). The above 6 sectors accounted for 66.15% of the total impact and also explained the direct impact by 90.76%. Steel Processing (S55) is another key sector due to its blue water use. In 2015, the 7 key sectors of blue water consumption in China explained 59.70% of the total impact and 86.94% of the direct effect. The key sectors for the use of grey water were Crop Cultivation (S1), Other Food Products (S18), Scrap and Waste (S83), Railway Freight Transport (S89), Highway Freight and Passenger Transport (S90), Water Freight and Passengers Transport (S92), Pipeline Transport (S95), and Health Services (S99) while the key sectors of grey water consumption of China in 2015 explained 81.28% of the total impact and 95.73% of the direct impact. In addition to the key sectors, there were a large number of direct-effect-based sectors, total-effects-based sectors, and non-key sectors.

7. In conclusion, please compare with previous studies. what are the similarities and differences?

Response：Since the raw data different researchers collect is not the same, there are different accounting results of China’s water footprint even with the same calculation method. For example, there are differences in terms of water footprint accounting results between this paper and existing literature on studies of water footprint for each sector in China[2]-[3][17]-[19][21], and there are differences among the literature itself. In the literature water footprint accounting in each region of China is based on the multi-region input-output model [1][25]-[27][32], the sum of water footprint in each region is not equal to China’s water footprint in this paper. Additionally, the focus of most literature on studies of China’s water footprint with input-output model is green water footprint and blue water footprint, while only a few [17] are about these three altogether: China’s green water, blue water and grey water footprint.

Several studies [2][21] stressed the impact of the virtual water trade and water footprint, but the focus of this paper is the analysis of the key sectors of the water resource consumption in China. Based on the cross-elasticity of the value added with the water consumption, we defined the DI indicator and TI indicator as methods to identify key sectors of the water consumption instead of considering the amount of water used for each sector alone. Therefore, not only do the key sectors identified in this paper require a large amount of water, but their economic growth also has a relatively significant impact on the water resource use of other sectors. Table 4 shows that the direct impact dominant sectors of China’s water consumption in 2015 are Vegetable Oil and Forage(S14), Slaughtering, Meat Processing, Eggs, and Dairy Products(S16), Prepared Fish and Seafood(S17), and Arts and Crafts Products(S81). Though these sectors used a relatively large amount of water (DI>0.003), the impact of their economic growth on the water consumption of other sectors was comparatively small (TI<0.003). Therefore, they were not ranked as key sectors of China’s water consumption in 2015.

The English language need to revise carefully.

Response: We have revised the English language carefully.
