# Supplementary material for: Identification of key sectors of water resource utilization in China from the perspective of water footprint
Source: PLoS One. 2020 Jun 22;15(6):e0234307. doi: 10.1371/journal.pone.0234307 (PMC7307777; doi:10.1371/journal.pone.0234307)
Supplement: Attachment — Submitted filename: Response.docx [file pone.0234307.s004.docx]

Dear editor and reviewers:

**Thank you for your comments on our manuscript entitled** “Identification of key sectors of water resource utilization in China from the perspective of the water footprint” (PONE-D-19-27097). **Those comments are very helpful for revising and improving our paper, as well as the important guiding significance to other research. We have studied the comments carefully and made corrections which we hope meet with approval. The main corrections are in the manuscript and the responds to the reviewers’ comments are as follows (the replies are highlighted in red).**

Editor: Please polish the language before the next submission.

Response: We have carefully revised the language again.

Reviewer #1: It is reasonable to study key sectors of water resource utilization in China. The authors completely answered the questions from the reviewer.

Response: Thank you.

There two questions required to response.

1.The English of the paper need to be revised again.

Response:We have carefully revised the language again.

2. The paper should cite more references. For example, Spatio-temporal analysis of driving factors of water resources consumption in China

Response: The revised version refers to the following papers:

[4]Long H, LinB, Ou Y, Chen Q(2019). Spatio-temporal analysis of driving factors of water resources consumption in China. Sci total environ 690: 1321-1330.

Reviewer #2: Compared with the previous version, the quality of this paper has improved greatly.

I recommend to accept this paper.

Response: Thank you.
