# Supplementary material for: Identification of key sectors of water resource utilization in China from the perspective of water footprint
Source: PLoS One. 2020 Jun 22;15(6):e0234307. doi: 10.1371/journal.pone.0234307 (PMC7307777; doi:10.1371/journal.pone.0234307)
Supplement: Attachment — Submitted filename: Response.docx [file pone.0234307.s005.docx]

Dear editor and reviewers:

**Thank you for your comments on our manuscript entitled** “Identification of key sectors of water resource utilization in China from the perspective of the water footprint” (PONE-D-19-27097). **Those comments are very helpful for revising and improving our paper, as well as the important guiding significance to other research. We have studied the comments carefully and made corrections which we hope meet with approval. The main corrections are in the manuscript and the responds to the reviewers’ comments are as follows (the replies are highlighted in red ).**

Replies to editorial comments:

PONE-D-19-27097R2
Identification of key sectors of water resource utilization in China from the perspective of the water footprint
PLOS ONE

Dear Dr Yue,

Thank you for submitting your manuscript to PLOS ONE. After careful consideration, we feel that it has merit but does not fully meet PLOS ONE’s publication criteria as it currently stands. Therefore, we invite you to submit a revised version of the manuscript that addresses the points raised during the review process. Especially, there are still a few writing errors, I suggest that the language should be polished by the language editing service.

We would appreciate receiving your revised manuscript by Jun 06 2020 11:59PM. When you are ready to submit your revision, log on to https://www.editorialmanager.com/pone/ and select the 'Submissions Needing Revision' folder to locate your manuscript file.

If you would like to make changes to your financial disclosure, please include your updated statement in your cover letter.

To enhance the reproducibility of your results, we recommend that if applicable you deposit your laboratory protocols in protocols.io, where a protocol can be assigned its own identifier (DOI) such that it can be cited independently in the future. For instructions see: <http://journals.plos.org/plosone/s/submission-guidelines#loc-laboratory-protocols>

Please include the following items when submitting your revised manuscript:

- A rebuttal letter that responds to each point raised by the academic editor and reviewer(s). This letter should be uploaded as separate file and labeled 'Response to Reviewers'.
- A marked-up copy of your manuscript that highlights changes made to the original version. This file should be uploaded as separate file and labeled 'Revised Manuscript with Track Changes'.
- An unmarked version of your revised paper without tracked changes. This file should be uploaded as separate file and labeled 'Manuscript'.

Please note while forming your response, if your article is accepted, you may have the opportunity to make the peer review history publicly available. The record will include editor decision letters (with reviews) and your responses to reviewer comments. If eligible, we will contact you to opt in or out.

We look forward to receiving your revised manuscript.

Kind regards,

Chuanwang Sun
Academic Editor
PLOS ONE

Response: we have polished the language again, and the attachment uploaded includes the Certificate of English Language Editing from LetPub ([www.letpub.com](http://www.letpub.com)).

In addition, this is an economics paper,so there is no laboratory protocols, but we performed the analysis with the help of MATLAB software, and the referred program is listed below.

>> clear

>> g1=zeros(1,99);

>> g2=zeros(1,99);

>> g3=zeros(1,99);

>> A=zeros(99,99);

s=zeros(1,99);

>> % g1, g2, and g3 input the share of green water, blue water, and gray water respectively; A enters the direct consumption coefficient; s enters the value-added coefficient

>> gg1=diag(g1);

>> gg2=diag(g2);

>> gg3=diag(g3);

>> I=eye(99);

>> ss=diag(s);

>> w1=gg1*inv(I-A')*ss;

>> w2=gg2*inv(I-A')*ss;

>> w3=gg3*inv(I-A')*ss;

>> w1i=sum(w1);

>> w1j=sum(w1,2);

>> w1ij=sum(sum(w1));

>> w2i=sum(w2);

>> w2j=sum(w2,2);

>> w2ij=sum(sum(w2));

>> w3i=sum(w3);

>> w3j=sum(w3,2);

>> w3ij=sum(sum(w3));

Reviewers' comments:

Reviewer's Responses to Questions


Reviewer #2: compared with previous studies, the quality of this paper has improved greatly.
I recommend to accept this paper.
Response: Thank you.

Reviewer #3: The article has been revised perfectly, the content is innovative, the angle is scientific and the method is feasible. Only one point: some fresh paper can be added as reference, eg:
Lu, S., Wu, X., Sun, H. et al. The multi-user evolutionary game simulation in water quality-based water source system. Environ Geochem Health (2019). https://doi.org/10.1007/s10653-019-00315-5.

Response: Thanks for the reviewer's comments. We have referred to this paper in the introduction section of the revision.
